# Supplementary material for: Auditory-motor synchronization varies among individuals and is critically shaped by acoustic features
Source: Commun Biol. 2023 Jun 21;6:658. doi: 10.1038/s42003-023-04976-y (PMC10284880; doi:10.1038/s42003-023-04976-y)
Supplement: Supplementary file 2 — Supplementary Material [file 42003_2023_4976_MOESM2_ESM.pdf]

Auditory-motor synchronization varies among individuals and is critically shaped by acoustic features

Cecilia Mares, Ricardo Echavarría Solana & M. Florencia Assaneo

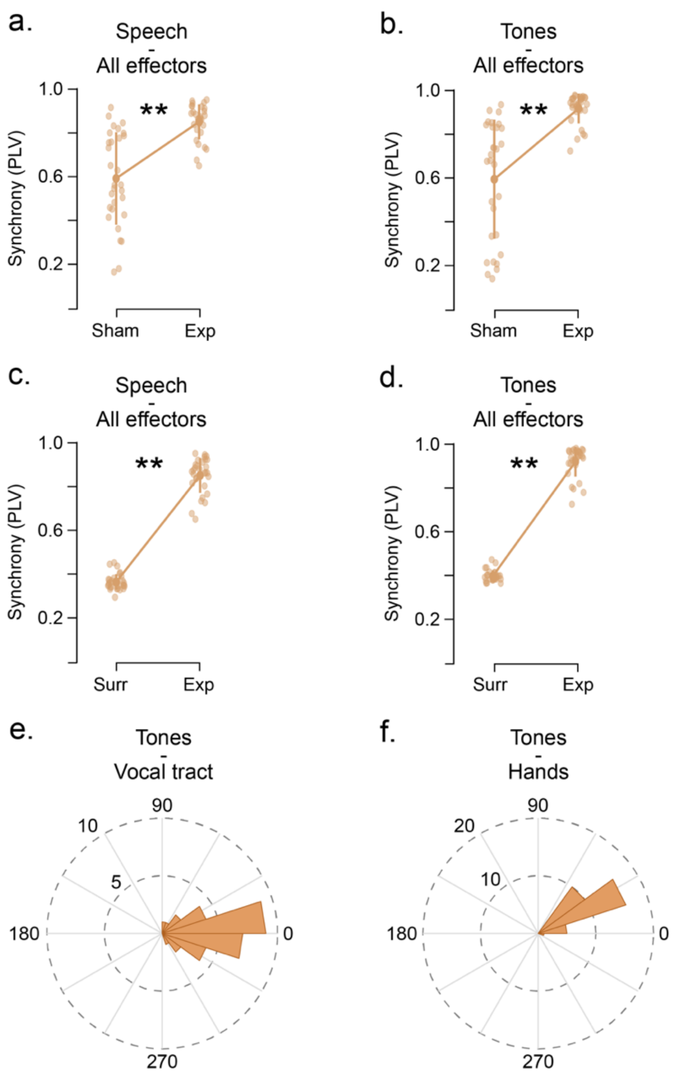

Supplementary Figure 1; High synchronizers' synchrony remains stable across stimuli. a&b. Sham synchrony (i.e., estimated with the whispering and clapping produced without auditory stimulation) compared to the one obtained during the main synchronization task. Average across effectors for speech like-stimulus and tones panel **a** and **b**, respectively (N=30). **c&d.** Surrogate synchrony (i.e., estimated between the sounds produced during the main task and a surrogate audio with a fixed rate of 4.3Hz) compared against the experimental synchrony (i.e., the one estimated between the sounds produced during the main task and the accelerated stimulus presented to the participants). Average across effectors for speech like-stimulus and tones panel **c** and **d**, respectively (N=30). Dots represent mean values, bars SD and **p<0.001. **e&f.** Rose plots illustrate the histogram of the mean phase lag between the produced and the perceived sounds. Lag between perceived tones and: whispered "tahs" in panel **e**, hands clapping in panel **f**. All panels relate to Experiment 1

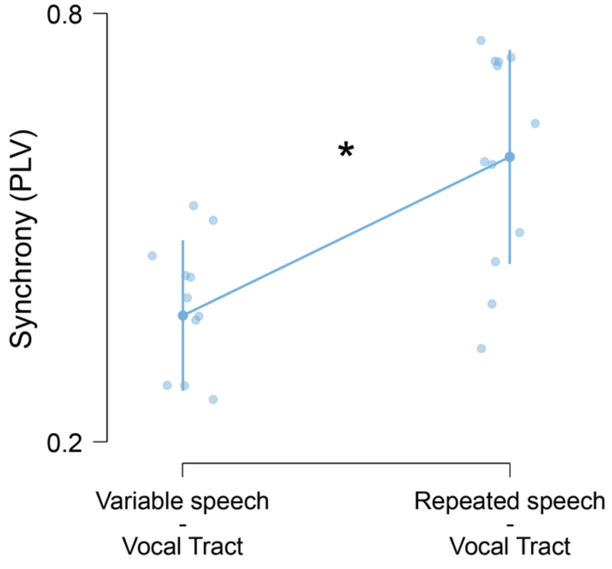

Supplementary Figure 2; The repetition of the same acoustic unit restores synchrony in low synchronizers. A group of low synchronizers (N=13) completed two extra synchronization tasks, both with the vocal tract as effector and differing in the acoustic stimulus. One task (Variable speech) used the same acoustic stimulus as experiment 1, which comprises a random concatenation of 16 different syllables. In the other task (Repeated speech), all syllables of the stimulus were replaced by the syllable "go". The plot displays the comparison between the synchronization values obtained for each task. Dots represent mean values, bars SD and *p<0.05.

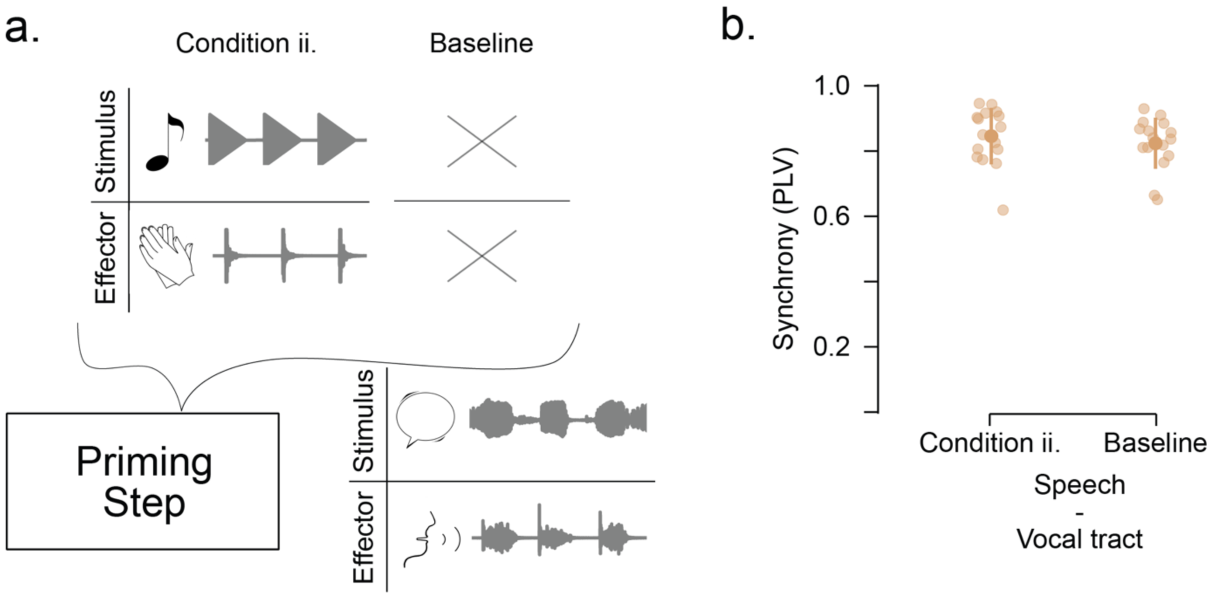

Supplementary Figure 3; Rhythmic priming does not benefit high synchronizers. a. Sketch of the condition ii (hands to tones synch) and baseline (no task), performed as priming steps, before the vocal tract to speech synchronization. **b.** Comparison between the vocal tract to speech synchrony value obtained after condition ii and baseline. No significant difference was found between them (t(15)=-0.979, p=0.343).

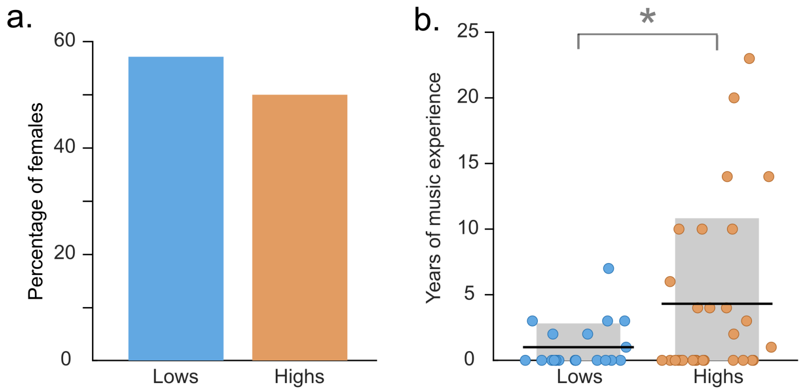

Supplementary Figure 4; Gender and years of music experience comparisons between groups. a. Percentage of female participants within both groups, no significant differences between groups in the gender distribution (Lows: N_{fem}/N_{tot}= 0.57; Highs: N_{fem}/N_{tot}= 0.50, Fisher's exact test p=0.77). **b.** Years of music experience within groups: number of years a participant played at least one musical instrument, no less than two hours per week (t(48)=-2.269, p=0.028). Blue/orange depict low/high synchronizers (N_{Low}= 21, N_{High}= 30), dots represent individual participants, black lines mean across groups, and gray region SD. *p<0.05.

Within Subjects Effects

Cases	F	p
Effector	1.804	0.185
Effector ✻ Group	8.288	0.006
Stimulus	68.077	< .001
Stimulus ✻ Group	29.283	< .001
Effector ✻ Stimulus	0.947	0.335
Effector ✻ Stimulus ✻ Group	1.1	0.299

Supplementary Table 1; Detailed statistical information from experiment 1 analysis. Repeated-measures ANOVA with two within-subject factors: effector (vocal tract vs. hands) and stimulus (syllables vs. tones) and synchrony group (high vs. low) as the between-subject factor.

Within Subjects Effects

Cases	F	p
Effector	3.657	0.062
Effector ✻ Group	7.923	0.007
Effector ✻ Gender	3.186	0.081
Effector ✻ Years music experience	0.691	0.41
Effector ✻ Group ✻ Gender	7.205	0.010
Stimulus	53.379	< .001
Stimulus ✻ Group	37.273	< .001
Stimulus ✻ Gender	4.584	0.038
Stimulus ✻ Years music experience	1.522	0.224
Stimulus ✻ Group ✻ Gender	5.268	0.026
Effector ✻ Stimulus	0.366	0.548
Effector ✻ Stimulus ✻ Group	2.27	0.139
Effector ✻ Stimulus ✻ Gender	3.803	0.057
Effector ✻ Stimulus ✻ Years music experience	1.029	0.316
Effector ✻ Stimulus ✻ Group ✻ Gender	1.27	0.266

Between Subjects Effects

Cases	F	p
Group	234.947	< .001
Gender	4.716	0.035
Years music experience	2.133	0.151
Group ✻ Gender	1.781	0.189

Supplementary Table 2; Detailed statistical information from experiment 1 analysis including gender and musical experience. Repeated-measures ANOVA, with two within-subject factors: effector (vocal tract vs. hands) and stimulus (syllables vs. tones). Synchrony group (high vs. low) and gender as between-subject factors and years of music experiences as covariate.
