# Supplementary material for: Auditory-motor synchronization varies among individuals and is critically shaped by acoustic features
Source: Commun Biol. 2023 Jun 21;6:658. doi: 10.1038/s42003-023-04976-y (PMC10284880; doi:10.1038/s42003-023-04976-y)
Supplement: Supplementary file 5 — Reporting Summary [file 42003_2023_4976_MOESM5_ESM.pdf]

nature portfolio

Corresponding author(s): M. Florencia Assaneo

Last updated by author(s): Apr 10, 2023

Reporting Summary

Nature Portfolio wishes to improve the reproducibility of the work that we publish. This form provides structure for consistency and transparency in reporting. For further information on Nature Portfolio policies, see our Editorial Policies and the Editorial Policy Checklist.

Statistics

For all statistical analyses, confirm that the following items are present in the figure legend, table legend, main text, or Methods section.

n/a	Confirmed	
☐	☒	The exact sample size (n) for each experimental group/condition, given as a discrete number and unit of measurement
☐	☒	A statement on whether measurements were taken from distinct samples or whether the same sample was measured repeatedly
☐	☒	The statistical test(s) used AND whether they are one- or two-sided<br>*Only common tests should be described solely by name; describe more complex techniques in the Methods section.*
☐	☒	A description of all covariates tested
☐	☒	A description of any assumptions or corrections, such as tests of normality and adjustment for multiple comparisons
☐	☒	A full description of the statistical parameters including central tendency (e.g. means) or other basic estimates (e.g. regression coefficient) AND variation (e.g. standard deviation) or associated estimates of uncertainty (e.g. confidence intervals)
☐	☒	For null hypothesis testing, the test statistic (e.g. F, t, r) with confidence intervals, effect sizes, degrees of freedom and P value noted<br>*Give P values as exact values whenever suitable.*
☒	☐	For Bayesian analysis, information on the choice of priors and Markov chain Monte Carlo settings
☒	☐	For hierarchical and complex designs, identification of the appropriate level for tests and full reporting of outcomes
☒	☐	Estimates of effect sizes (e.g. Cohen's d, Pearson's r), indicating how they were calculated

Our web collection on statistics for biologists contains articles on many of the points above.

Software and code

Policy information about availability of computer code

Data collection	Google Forms was used to get the demographic data from participants. MATLAB R2020b and Psychotoolbox v3.0.17 were used to present stimuli and record all participant's responses.
Data analysis	Data processing and analyses were conducted using MATLAB R2020b and Jasp v0.14.1.

For manuscripts utilizing custom algorithms or software that are central to the research but not yet described in published literature, software must be made available to editors and reviewers. We strongly encourage code deposition in a community repository (e.g. GitHub). See the Nature Portfolio guidelines for submitting code & software for further information.

Data

Policy information about availability of data

All manuscripts must include a data availability statement. This statement should provide the following information, where applicable:

- Accession codes, unique identifiers, or web links for publicly available datasets
- A description of any restrictions on data availability
- For clinical datasets or third party data, please ensure that the statement adheres to our policy

The data supporting the findings of this study are available as Supplementary Data. All other data are available from the corresponding author.

Research involving human participants, their data, or biological material

Policy information about studies with human participants or human data. See also policy information about sex, gender (identity/presentation), and sexual orientation and race, ethnicity and racism.

Reporting on sex and gender	Neither sex nor gender was considered in the study design. Gender was self-reported. The first group included 27 females and 24 males. A second group included 10 females and 6 males. The third and last group included 14 females and 17 males. A repeated measures ANOVA was conducted, including gender as a between-subject factor for the results obtained for Experiment 1.
Reporting on race, ethnicity, or other socially relevant groupings	No race, ethnicity, or other socially relevant variables were included in this work.
Population characteristics	The research sample was composed mainly of UNAM graduates. Since this study was performed during a pandemic, it was far more practical to recruit students who were indeed attending the university facility. First group: n=51; 27 females; mean age, 27 years; range, 21-37 years. Second group: n=16; 10 females; mean age, 27 years; range, 23-40 years. Third group: n=31; 14 females; mean age, 26 years; range, 23-40 years.
Recruitment	Participants were recruited by an announcement with the invitation to participate in the experiment which was placed around the UNAM facility.
Ethics oversight	Ethics Committee of the Instituto de Neurobiología of Universidad Nacional Autónoma de México (protocol 096.H)

Note that full information on the approval of the study protocol must also be provided in the manuscript.

Field-specific reporting

Please select the one below that is the best fit for your research. If you are not sure, read the appropriate sections before making your selection.

☒ Life sciences ☐ Behavioural & social sciences ☐ Ecological, evolutionary & environmental sciences

For a reference copy of the document with all sections, see nature.com/documents/nr-reporting-summary-flat.pdf

Life sciences study design

All studies must disclose on these points even when the disclosure is negative.

Sample size	No sample-size calculation was performed. The sample size was chosen in line with other studies with similar experimental procedures. See for example:<br>Repp, B., & Jendoubi, H. (2009). Flexibility of temporal expectations for triple subdivision of a beat. Advances in Cognitive Psychology, 5(1), 27–41. https://doi.org/10.2478/v10053-008-0063-7<br>Grahn, J. A., & Brett, M. (2007). Rhythm and Beat Perception in Motor Areas of the Brain. Journal of Cognitive Neuroscience, 19(5), 893–906. https://doi.org/10.1162/JOCN.2007.19.5.893<br>Orpella, Joan, et al. "Differential activation of a frontoparietal network explains population-level differences in statistical learning from speech." PLoS biology 20.7 (2022): e3001712.
Data exclusions	Originally, 5 extra participants participated in this study but were removed because of an exclusion criterion, which was pre-established by Lizcano-Cortés, et al., 2022. It refers to participants who had not completed the synchronization tasks successfully (i.e., they set the volume too loud such that the stimulus leaked in the recording, the rate of the motor gesture was equal to or less than 2 Hz or participants remained silent for more than 3 sec).
Replication	Several analyzes were performed to ensure the validity of the presented results and different cohort of participants were evaluated validating the obtained outcomes.
Randomization	No prior experimental groups were defined. Our group distinction has been done based on a clustering algorithm run on the collected data.
Blinding	Investigators were blind to group allocation

Reporting for specific materials, systems and methods

We require information from authors about some types of materials, experimental systems and methods used in many studies. Here, indicate whether each material, system or method listed is relevant to your study. If you are not sure if a list item applies to your research, read the appropriate section before selecting a response.

Materials & experimental systems

n/a	Involved in the study
☒	☐ Antibodies
☒	☐ Eukaryotic cell lines
☒	☐ Palaeontology and archaeology
☒	☐ Animals and other organisms
☒	☐ Clinical data
☒	☐ Dual use research of concern
☒	☐ Plants

Methods

n/a	Involved in the study
☒	☐ ChIP-seq
☒	☐ Flow cytometry
☒	☐ MRI-based neuroimaging
